# *CREBBP* and *WDR 24* Identified as Candidate Genes for Quantitative Variation in Red-Brown Plumage Colouration in the Chicken

**DOI:** 10.1038/s41598-020-57710-7

**Published:** 2020-01-24

**Authors:** J. Fogelholm, R. Henriksen, A. Höglund, N. Huq, M. Johnsson, R. Lenz, P. Jensen, D. Wright

**Affiliations:** 1 AVIAN Behavioural Genomics and Physiology Group, IFM Biology, Linköping University, Linköping, 58183 Sweden; 2 ITN Dept of Science and Technology, Linköping University, Linköping, 58183 Sweden; 3 The Roslin Institute and Royal (Dick) School of Veterinary Studies, The University of Edinburgh, Midlothian, EH25 9RG Scotland United Kingdom; 4 Department of Animal Breeding and Genetics, Swedish University of Agricultural Sciences, Box 7023, 750 07 Uppsala, Sweden

**Keywords:** Evolutionary genetics, Gene expression, Quantitative trait

## Abstract

Plumage colouration in birds is important for a plethora of reasons, ranging from camouflage, sexual signalling, and species recognition. The genes underlying colour variation have been vital in understanding how genes can affect a phenotype. Multiple genes have been identified that affect plumage variation, but research has principally focused on major-effect genes (such as those causing albinism, barring, and the like), rather than the smaller effect modifier loci that more subtly influence colour. By utilising a domestic × wild advanced intercross with a combination of classical QTL mapping of red colouration as a quantitative trait and a targeted genetical genomics approach, we have identified five separate candidate genes (*CREBBP, WDR24, ARL8A, PHLDA3, LAD1*) that putatively influence quantitative variation in red-brown colouration in chickens. By treating colour as a quantitative rather than qualitative trait, we have identified both QTL and genes of small effect. Such small effect loci are potentially far more prevalent in wild populations, and can therefore potentially be highly relevant to colour evolution.

## Introduction

Plumage colouration in birds is involved in a wide range of functions from camouflage^1^, to sexual signaling^2^ and species recognition^3^. The genetics of mammalian coat colour has been studied extensively, with many genes of major effect identified^4^. Feather colour is more complex than mammalian hair colour, with multiple colours and patterns possible on a single feather (intra-feather patterning)^5^. Similarly, patterning may also be present more broadly across distinct body regions^5^. The evolutionary importance of feather colouration can be seen most prominently in their role in signalling a variety of traits relating to sexual selection^6,7^. For example, carotenoid pigmentations can reflect increased immunocompetence^8^, parasite resistance^9^, and foraging efficiency^10^, eumelanin pigmentation can signal increased body condition^11,12^, testosterone level^13^, immunocompetence^14^ and parasite load^15^, whilst phaeomelanin can signal increased male reproduction^16^ and survivability^17^. In this way, understanding the genes underlying colour morphology can have ramifications for a number of evolutionary topics.

There are three main types of pigmentation in feathers – melanin, carotenoids and porphyrins^18^. In the chicken carotenoid colouration is rare, and has been found to be absent in the feathers of both domestic chicks^19^ and adults, as well as in wild Red Junglefowl (RJF) adults^20^. The main form of pigmentation in chickens is via melanin^5^. Melanocytes are derived from unpigmented melanoblasts, and begin to synthesise melanin around embryonic stage E7-E8. Melanocytes can give rise to either eumelanin (creating black or grey colouration) or phaeomelanin (creating brown, red or yellow pigmentation)^21^. Importantly for understanding their genetic architecture, melanins are not environmentally constrained, unlike carotenoids^22^.

There are numerous genes implicated in plumage colour variation. Examples of causative genes for pigmentation include well studied colour determining genes such as *MC1R* (involved in the melanin pathway where its activation leads to the production of black eumelanin^23^) and triggers the expression of *MITF*, which activates the enzyme Tyrosinase^24^, a rate limiting step in the melanin synthesis pathway. *MC1R* has been implicated in the control of coat colour in a number of species^4,25–28^. Single mutations in *MC1R* in the lesser snow goose and arctic skua also explain nearly all the melanin differentiation in these two bird species^29^. In barn owls a single substitution in *MC1R* has been shown to explain up to 30% of the variation in three melanin-based colour traits (the presence of reddish brown, and the number and size of black dots on the plumage)^30^. The *ASIP* gene (agouti-signalling-protein) is an antagonist of *MC1R* and acts to nullify it, with loss-of-function of *ASIP* leading to the production of eumelanin, whilst in the case of *MC1R* loss of function leads to an increase in phaeomelanin (see review in^31^). A mutation upstream of *ASIP* has been implicated in the yellow colouration in Japanese Quail^32^. Tyrosinase and tyrosine-related proteins also interact with *MITF* and can lead to hypoalbinism in particular^4^. Examples of other mutations affecting phaeomelanin also include *sut (subtle grey)*, a mutation in the *SLC7a11* gene that reduces phaeomelanin production via lysosome-related organelles^33^, while the *Darkbrown* phenotype in chickens reduces black pigmentation and increases red phaeomelanin, and is caused by a deletion upstream of the gene *SOX10*^34^.

The above genetic variants can all be considered of major effect, in that they cause albinism, black plumage, etc in almost a Mendelian fashion. The above polymorphisms are responsible for at least 30% of the variation in the traits in the above examples (though often a variety of epistatic interactions also occur – see^35^). However, to the best of our knowledge, smaller effect loci that have less extreme modifier effects, in essence quantitative trait loci for plumage colour, have yet to be identified (such QTL typically have effects sizes of 3–5% of the variation in the trait). In natural conditions, it may be of greater importance to more subtly modify coat colouration, which is why many of the genes (though not all- see^29,30^) with more extreme phenotypic effects are identified in laboratory or domestic populations, where natural selection is essentially removed or greatly diminished.

Domesticated animals are a classical example of extreme variability in pigmentation within species. A reduction in pigmentation and colouration variability is usually considered as one of the key components of the domestication syndrome^36^, however artificial selection has also led to novel colour phenotypes in domesticated animals^37–39^. In the case of coat colouration, domestic animals are markedly different to their wild ancestors. This is for instance the case with chickens, where the wild ancestor the Red Junglefowl has a wide range of plumage colours ranging from dark red/ brown to light orange within a single individual. In the case of domestic chicken breeds, and in particular layer and broiler breeds, plumage is often restricted to either white or brown colouration. For example, many of the major layer breeds are either white (White Leghorn, White Dorking, Bresse Gauloise, for example) or brown (Isa Brown, Hyline Brown, Lohman Brown, Rhode Island Red, amongst others), though breeds with more diversity in colouration do exist (the Australorp is generally black for example, though blue and white variants also exist). Certainly as a comparison, the White Leghorn layer birds are in stark contrast to the colourful Red Junglefowl in that they are completely white and display no sexual dichromatism.

To identify genes that regulate quantitative variation in the intensity of red-brown colouration, we utilized an advanced intercross between wild Red Junglefowl and domestic White Leghorn (WL) chickens. The Red Junglefowl are elaborately coloured and display some of the widest range of colour variation seen in the chicken (possessing dark red, light red and orange coloured plumage in a variety of hues). This means that a wide range of red-brown colour variation is seen in these birds, with both light red and dark red plumage present. In contrast the White Leghorn birds are fixed for the dominant white locus and are pure white in colour. The intercross birds display an enormous range of plumage colouration with regards to both hue and intensity, reflecting the large differences seen between the parental types used. Genetically the advanced intercross gives far smaller confidence intervals for detected Quantitative Trait Loci (QTL) than standard F_2_ intercrosses^40^. The study presented here utilises multiple generations for this analysis. A large-scale QTL scan was performed using the F_8_ generation, whilst further targeted expression QTL (eQTL) studies were performed using the F_10_ and F_12_ generations to assess candidate genes identified in the QTL scan. By using a combination of targeted genetical genomics (whole genome transcriptomics of targeted individuals) to simultaneously map eQTL and correlate gene expression with intensity of red-brown colouration, we identify five putatively causal genes affecting quantitative variation in this plumage colouration trait in the chicken.

## Results

### QTL analysis

QTL analyses were performed using both peak red intensity and overall red intensity in the F_8_ birds (n = 380). Three separate QTL were identified for peak intensity on chromosomes 2 at 149 cM, chromosome 10 at 176 cM, and chromosome 14 at 207 cM (see Table 1). In the case of the loci on chromosomes 2 and 10, the Red Junglefowl genotype led to an increase in red intensity, whilst for the chromosome 14 locus the White Leghorn genotype led to an increase in red intensity. An epistatic interaction was found between the loci on chromosomes 2 and 10 (see Supplementary Table 1 for a break-down of these interaction effects). This effect was mainly based on additive × additive and additive × dominance effects (see Supplementary Table 1), though the individual loci were significant prior to the interaction being incorporated (see Supplementary Table 2 for the QTL effects without the interaction). Four separate QTL were identified for overall red intensity on chromosomes 2 (at 81 cM), 11 (at 73 cM), 15 (at 148 cM) and 26 (at 0 cM), see Table 1. These QTL were significant as two epistatic pairs, with an interaction between the loci on chromosomes 2 and 11, and an interaction on chromosomes 15 and 26. Once again, for two of the loci (those on chromosomes 2 and 15) the Red Junglefowl genotype led to an increase in red intensity. With the epistatic interaction between the QTL on chromosomes 2 and 11, the additive effect was magnified, with a White Leghorn genotype at both QTL giving the biggest increase in colour score (i.e. a whiter colour, or less red intensity, see Supplementary Table 1 and Supplementary Fig. 1). The other pair of epistatic loci on chromosomes 11 and 26 mainly acted through dominance rather than additive variation. In particular, an over-dominance interaction was seen with a homozygous Red Junglefowl genotype on the chromosome 15 locus and a homozygous White Leghorn genotype on the chromosome 26 locus leading to the biggest decrease in colour score (i.e. a redder phenotype), see Supplementary Fig. 1.

**Table 1:** Results from the QTL scan of the two phenotypes(red spot intensity and average red red) in the initial F_8_ mapping population.

trait	chr	position	LOD	R2	add +/− s.e.	dom +/− se	lower CI	upper CI	lower_marker	pos (bp) lower	upper_marker	pos (bp) upper	covariates	interaction
overall_red_intensity	2	81	9.0	6.8	7.5 +/− 3.2	11.2 +/− 4.6	70	93	rs14133982	6928346	rs14139143	11611159	sex, batch, PC2, PC3	2@81.0:11@73.0
overall_red_intensity	11	73	9.9	5.6	1.4 +/− 8.1	15.4 +/− 11.9	61	80	rs14961831	7419207	rs14025092	10674175	sex, batch, PC2, PC3	2@81.0:11@73.0, sex:11@73.0
overall_red_intensity	15	148	6.5	4.8	7.2 +/− 4.2	8.8 +/− 9.1	125	169	rs15023850	9129598	rs15025752	11115930	sex, batch, PC2, PC3	15@148.0:26@0.0
overall_red_intensity	26	0	8.7	6.5	−7.4 +/− 2.8	12.7 +/− 3.8	0	5	rs13724830	213621	rs15466096	1092863	sex, batch, PC2, PC3	15@148.0:26@0.0
spot_red_intensity	2	149	7.1	6.6	0.06 +/− 0.01	0.02 +/− 0.02	96	164	rs15060526	8300941	rs15069282	18353968	sex, batch, PC1, PC3	2@149.0:10@176.0
spot_red_intensity	10	177	6.3	5.8	0.12 +/− 0.04	0.05 +/− 0.05	162	208	rs14947769	11123643	rs14951592	15091654	sex, batch, PC1, PC3	2@149.0:10@176.0, 10@176.0:sex
spot_red_intensity	14	208	5.8	5.4	−0.09 +/− 0.02	−0.05 +/− 0.03	172	223	rs14076550	8493227	rs15002638	13224771	sex, batch, PC1, PC3	

QTL locations (in cM), Lod scores, effect sizes (r^2^), additive and dominance values, confidence intervals (as per a 1.8 lod drop method), covariates (described in detail in the methods section) and interactions are all shown.

### Overlap with known colour Loci

To check whether any of the previously detected classical colour loci also contribute to small-scale variation, the detected QTL were checked for the presence of these genes. None of the QTL were found to overlap any previously identified colour loci, with the following genes checked: *SOX10, Tyr, SLC45a2, MITF, MC1R, MLPH, ASIP, SLC34a2, PMEL, SLC7a11*.

### Functional validation via targeted eQTL analyses

The loci that were identified using the initial genome-wide F_8_ scan were then assessed using microarray gene expression data obtained from growing calami in adult F_10_(n = 12) and F_12_ (n = 12) individuals. We correlated the expression levels to the relative intensity of red colouration with all genes (a total of 875 probesets, see Supplementary Table 1) located within the confidence intervals of the seven QTL regions. After multiple testing corrections (FDR, p = 0.05) were applied we found 76 probesets that were correlated with the peak intensity of red colouration (FDR adjusted p-value < 0.05, See Supplementary Table 2).

In order to further narrow down the list of potential candidate genes we performed an eQTL analysis using the probesets that correlated with colour using a set of targeted markers located within the colour QTL regions. We detected five eQTL genes (six probesets) at a genome-wide suggestive level (including a multiple testing correction for the total number of genes tested), two for genes associated with overall red intensity and four that are associated with the peak red intensity (see Table 2). All were trans-eQTLs, being controlled from a region on chromosome 10 (rs 14949856 @ 15038239 bp).

**Table 2:** Summary of candidate genes with both a significant correlation with red intensity and a significant eQTL.

Gene	logFC	AveExpr	t	P,Value	adj,P,Val	B	eQTL origin	eQTL marker snp	additive effect ( +/− SE)	dominance effect ( +/− SE)	% of variation explained	lod	Gene location
NM_001030628_WDR24	4.52	11.49	3.20	0.0036	0.0466	−1.83	Chr 10	Gg_rs14949856	0,9190 (0,3162)	1,9981 (0,4790)	51.12	3.73	14
ENSGALT00000031449_PHLDA3	4.04	8.07	3.12	0.0045	0.0477	−1.97	Chr 10	Gg_rs14949856	0,8264 (0,2938)	1,7414 (0,4451)	48.37	3.45	26
ENSGALT00000031450_LAD1	2.87	13.42	2.85	0.0085	0.0499	−2.51	Chr 10	Gg_rs14949856	0,6549 (0,2196)	1,2526 (0,3327)	48.06	3.41	26
ENSGALT00000012587_CREBBP	4.24	10.02	3.06	0.0051	0.0482	−2.13	Chr 10	Gg_rs14949856	0,8353 (0,3221)	1,8305 (0,4879)	45.71	3.18	14
NM_001012868_ARL8A	3.04	12.40	2.99	0.0061	0.0492	−2.23	Chr 10	Gg_rs14949856	0,5042 (0,2297)	1,3767 (0,3480)	45.82	3.19	26
ENSGALT00000000410_LAD1	4.39	11.76	2.90	0.0075	0.0492	−2.40	Chr 10	Gg_rs14949856	0,9748 (0,3519)	1,8517 (0,5331)	44.18	3.04	26

The table includes the log of Fold change for the probe in question, t value of the gene expression, the adjusted p-value and the B value for the correlation between expression levels and colour score. In addition, it has the chromosomal location of the eQTL marker (plus the marker name), the additive and dominance effects of the eQTL, the % of variation explained by the eQTL (r-squared value), lod score and the location of the gene itself.

### A network of trans-eQTL between colour QTL

**Figure 1:**
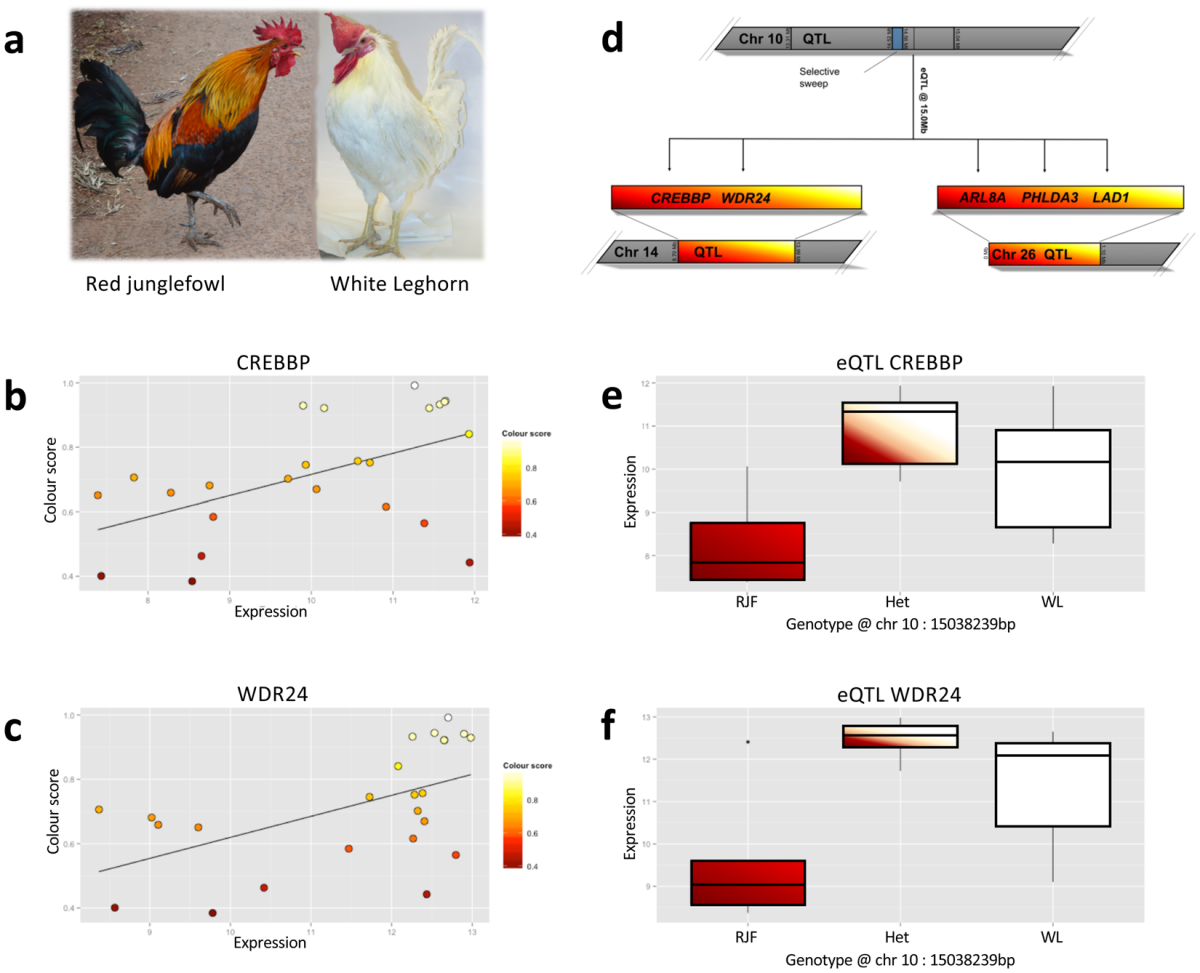
An overview of the main findings from the colour/gene expression correlation as well as the eQTL mapping. (**a)** This shows a representative picture of the two extreme ends of our phenotype, note that since our individuals come from an intercross population the majority of them will have an intermediate phenotype, which commonly manifests as a white chicken that retain different parts of the red patterning. (**b,c)** Shows the correlation between the colour score and the amount of gene expression for two of the candidate genes, *CREBBP* and *WDR24* which are both located on chr 14. (**d)** Is a visual representation of the trans-eQTL effect originating on chr 10 that influences gene expression of a total of five genes located on chr 14 and chr 26. (**e,f)** Boxplots that show the link between the genotype at chr 10: 15038239 bp and gene expression for *CREBBP* and *WDR24* respectively.

We see a trans regulation of candidate gene expression, with a locus on chromosome 10 controlling multiple target sites. The most significant trans-eQTL effect originates from chromosome 10 and controls two genes on chromosome 14 *(CREBBP* and *WDR24)*, and three genes on chromosome 26 (*ARL8A*, *PHLDA3*, and *LAD1*), see Fig. 1. There is also a selective sweep^41^ located adjacent (~500 kb) to the chromosome 10 eQTL marker and also within the confidence interval for the colour QTL identified in the F_8_ population.

## Discussion

The aim of this study was to identify genes and genetic loci that affect red-brown colouration in the chicken. By treating colour as a quantitative trait, as opposed to looking for solely discrete classes, we have identified a total of seven small-effect QTL that until now have been overlooked. Furthermore, by combining this with a targeted genetical genomics approach in a subsequent population we have identified five candidate genes affecting the intensity of red colouration. These genes would be unlikely to have been identified using more classical (presence/ absence of major genes) linkage methods. This is unsurprising given that the average effect size was around 7% in our study, whilst the high degree of epistasis between loci would have meant they would have been extremely hard to pinpoint using single scan approaches. In particular the chromosome 10 locus appears to have a strong effect, controlling loci on chromosomes 14 and 26. It therefore appears that this locus has trans acting effects in combination with epistasis. This locus also contains a selective sweep that is fixed in domestic layer birds (the majority of which are white), yet segregating in wild Red Junglefowl. A total of 408 sweeps are present throughout the genome, with five sweeps present on chromosome 10, with each sweep being on average 40 kb in length^41^. However, whether this sweep contains the causal element responsible for colour variation remains to be validated.

Several genes have been identified as affecting colour in the chicken, however these are all major-effect genes that have been identified using a classical linkage/single gene approach. In the chicken, the Dominant White locus (on chromosome 33, an allele of the *PMEL* gene)^42^, the classic extended phenotype (E) linked to *MC1R* on chromosome 11 at 19 Mb^43,44^, and the Dark Brown (Db) locus on chromosome 1 (linked with *SOX10*)^45^ all affect pigmentation (white/black in the case of Dominant White and E, and red in the case of Dark brown), whilst sex-linked barring (on chromosome Z) and the *EDNRB2* (panda) locus in quails^46^ primarily affect patterning. Despite these examples, studies of pigmentation inheritance in crosses imply that many more genes have yet to be identified. Specifically, to examine buff, brown and red colouration, crosses have been made with a variety of breeds, including Junglefowl × Buff Minorca and Rhode Island Red breeds^47^, Brown and Light Brown Leghorn breeds^48^, Dark Cornish and Black Breasted Red^49^, and Villafranquina birds^50^. These studies reveal a complex pattern of inheritance and multiple modifier loci exist that affect phaeomelanin. For example with the buff phenotype, four autosomal factors were proposed, named ginger, mahogany, dilute and champagne blond^47^, whilst many studies noted many variations in the intensity of the red colouration present, even when segregating for a known major effect gene^48,50^. The wide variety of phenotypes that rapidly emerge indicate that multiple epistatic interactions and numerous genes of small effect is the norm, yet such modifier loci have yet to be identified.

The QTL that we report here overlap none of these previously identified major effect loci. The genes and QTL are all novel loci (as affecting colour), though in the case of the QTL on chromosome 11, this is around 4 Mb downstream of the *MITF* gene. It is possible that this locus is a modest transcription factor or modifier element affecting the *MITF* gene, however no correlation was found between any of the colour measurements and *MITF* expression. Interestingly, the linkage between this locus and *MITF* may have even caused the effects to be erroneously ascribed to *MITF* alleles. When considering the classical studies it is perhaps logical that these modifier loci are discrete to the genes identified previously. For example Dominant White is known to be ineffective against red phaeomelanin, whilst mutations in the silver allele for sex-linked silver (*SLC45A2*) are incompletely dominant to the wild-type and highly influenced by modifying genes^45^. Our study indicates that by concentrating solely on such major genes, which are easier to identify, much variation is missed.

Of the candidate genes identified, particular interest comes from the interaction and correlation seen between the gene *CREBBP* and red intensity. *CREBBP* is controlled by the trans-acting eQTL locus on chromosome 10 that also contains the selective sweep. *CREBBP* binds to *CREB*, whilst *CREB* itself is a transcription factor strongly involved in melanin synthesis, principally by binding and activating *MITF* via the cAMP response element^51^. In turn *MITF* has a role in activating tyrosinase^52^, which in turn oxidates tyrosine to DOPA and affects which type of melanin (eu- or phaeo-melanin) is produced^53^. Given this role of *CREB* and its close interplay with *CREBBP*, it therefore represents a strong candidate as a small-effect modifier of red-brown colouration via melanogenesis to alter phaeomelanin colouration. Currently, *CREBBP* has primarily been found to be involved with neuronal growth and development^54,55^, so a role in feather colour expression is relatively novel. Of the other candidate genes identified, *WDR24* is a master regulator of lysosome function. Given one function of lysosomes is to regulate and degrade pigment molecules (for example a mutation in the *SLC7a11* gene reduces phaeomelanin production via lysosome-related organelles^33^), this also represents another excellent candidate gene. Similarly, the gene *ARL8A*, an acetylated Arf-like GTPase, is localized to lysosomes and affects their mobility^56^. Of the remaining genes, *PHLDA3* is implicated in tumour suppressor function, is a target of p53 and also impedes somatic cell reprogramming^57^, whilst *LAD1* encodes ladinin-1, a collagenous anchoring filament protein that is involved with the maintenance of cohesion at the dermal-epidermal boundary^58^, and defects in this gene are related to linear IgA disease, an autoimmune blistering disease^59^.

In summary, by treating the intensity of red colouration as a purely quantitative trait and utilising a variety of advanced intercross generations and targeted gene expression profiling we are able to identify multiple small-effect loci that interact to determine the red colouration present in Red Junglefowl. Using a targeted genetical genomics approach we are able to identify several high quality candidate genes that appear to have direct relevance to the melanogenesis pathway and can thus modify overall red intensity. However, one caveat is that the relatively low number of birds used in the targeted genetical genomics approach means that weaker effect genes will be missed (i.e. those with a weaker correlation, or non significant eQTL), therefore other candidates may still be present in the regions. Similarly, the genes identified are only putative, and full functional assays are required to verify that these genes are indeed causal to the phenotype. As far as we are aware this is the first study to identify QTL and candidate genes that can more subtly modify the intensity of red colouration in feathers, with such small effect loci being potentially far more prevalent in wild populations.

## Methods

### Study animals

**Figure 2:**
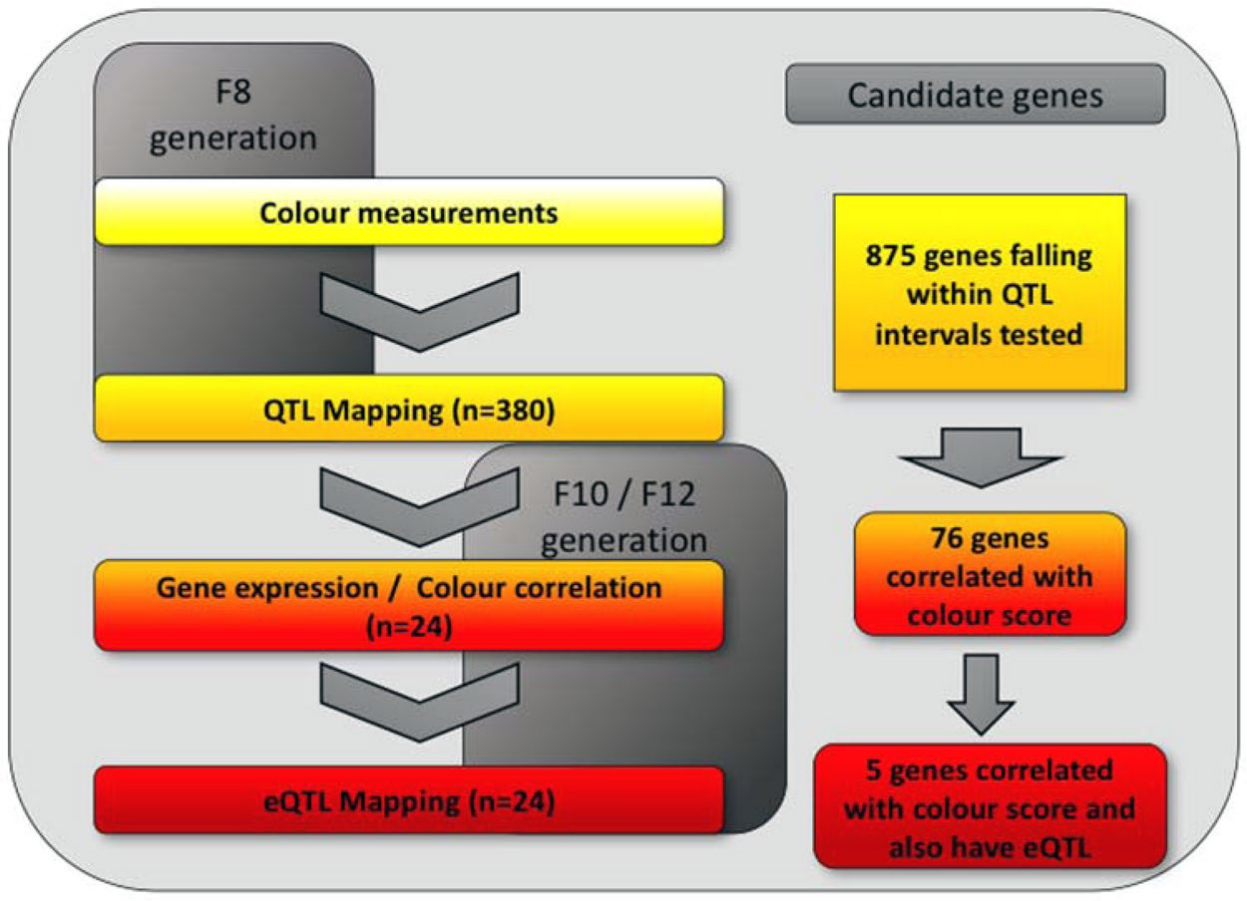
A flowchart of the experimental set-up used. The number of birds used in the F_8_ generation and the F_10_ and F_12_ generations are shown, along with what experiment they were used for (QTL or eQTL), as well as the number of candidate genes generated at each step.

The animals used in this study come from three separate generations (F_8_, F_10_ and F_12_) of an intercross between a Red Junglefowl male and three females from a White Leghorn selection line (WL-L13) maintained from the 1960s at first SLU, Uppsala and subsequently in Linköping, Sweden (since 2006). These four parental birds were used to generate 41 F_1_ individuals that were in turn used to generate 811 F_2_ individuals. From the F_3_ generation to the F_7_ generation, the intercross was maintained at ~100 individuals per generation, until the F_8_ generation, which consisted of a mapping population of 572 individuals reared in 6 batches, with wings saved from 380 of these birds. The F_10_ and F_12_ generations were also of approximately 100 individuals each, with 12 F_10_ and 12 F_12_ individuals used in this study. The selection of these birds (from the F_10_ and F_12_ generation) was based on those displaying red-brown colouration, with only these 24 birds utilised in this study. Food and water was supplied *ad libitum*, see^60^ for further details of housing and the intercross. A flow-chart of the birds used in the experiment, and which generation they are from is provided in Fig. 2. Animals were killed by cervical dislocation.

### Ethics approval

The study was approved by the local ethical committee of the Swedish National Board for Laboratory Animals. All experiments were performed in accordance to all relevant guidelines and regulations.

### Colour phenotyping

Wings were assessed for both the intensity and coverage of red-brown colouration in all the animals tested. The wing was selected for several reasons. We firstly observed that a large degree of colour variation was present on the wing, but importantly it was far easier to accurately assess overall degree of colouration in a far more repeatable fashion and with a high degree of accuracy due to the wing being flat and therefore enabling it to be photographed in a highly standardised fashion (see below). This enabled far more precise inter-individual comparisons to be made, which is essential for genetic mapping experiments. In terms of ecological relevance, the Red Junglefowl more frequently perform wing displays, implying that it is also used for signalling in natural populations^61^. Wings were removed post-mortem from individuals after slaughter at 212 days in the case of the F_8_ individuals. Each wing was photographed using a tripod mounted NIKON D5500 with a 40 mm lens using a LF-PB-3 (Falcon Eyes) light tent and two fixed 24Watt DULUX L (Osram) lights mounted to brackets to ensure identical lighting conditions were used for each wing. A standardized blue background was used for each shot, whilst an X-rite colour checker chart (with both pure white and red coloured squares) was included in each photograph to normalize colour levels. Wing photos were taken on the blue background, and a corresponding image was taken of the blank background without the wing present. Raw image quality was used for subsequent calculations. Two separate phenotypes were extracted from each wing, peak and overall red intensity. Peak intensity was measured by selecting an area of approximately 1 cm^2^ (~14000 pixels) in the area with the strongest representative red intensity observed by eye in Adobe Photoshop CS6 and recording the median value from the red RGB channel. This value was then divided by the value measured from the ‘true’ red on the colour chart, to give a normalized peak red intensity for each wing. It is worth noting that since white is composed of full saturation in all channels this red value is negatively correlated with red colour, ie the darker red the feather is the lower the relative colour score will be and vice versa. The second phenotype uses the average red intensity measured across each wing. This was performed using the following steps:

#### Colour correction

The raw sensor measurements (pixel values without any of the usual processing of the camera) were extracted from the image file using the program dcraw (see http://www.cybercom.net/~dcoffin/dcraw/ for the original version of the program and http://www.guillermoluijk.com/tutorial/dcraw/index_en.htm for a tutorial describing the program). For each of the images the location of the white patch of the colour checker with the highest intensity was automatically detected and the mean RGB vector over all pixels in this patch was computed. The colour vectors of all pixels in the image were then scaled (and converted to 16-bit unsigned integer) such that the resulting pixels in the white patch had all the same grey values. After this step all colour variations due to varying illumination and camera conditions were eliminated and all images could be compared.

#### Geometric correction and compilation of the wing mask

After normalization, colour correction registration points (essentially two crosshairs on the left and the right side of the blue background) were automatically detected in both images of an image pair. The locations of these registration points were then used to compute the geometric transformation that aligned the images without the wing with the image with the wing. Pixelpairs with blue colour in the background image and non-blue colour in the wing image define a mask indicating the location of the wing.

#### Extraction of red pixels

The mask computed in the previous step is used to set all non-wing pixels to a black background colour. Next the colour vectors in the masked image are converted from the RGB colour space to the HSV colour space using the Matlab function rgb2hsv. In this HSV the colour of a pixel is described by three values: H is the hue (described by an angle), S is the saturation described by an interval and finally V is the value, which is a non-negative number describing the lightness. In the application only red/brown pixels are of interest. The corresponding region in colour space was defined as the region where the hue value was between 0.6 and 0.7 and the saturation value was greater than 0.75. Both the hue and the saturation values are located in the [0,1] interval. This procedure thus isolates all the red/brown patterned feathers from the rest of the wing. After the pattern isolation, the red channel of the entire resulting trimmed image was measured in Photoshop. Therefore this second phenotype takes the median red intensity of every red/brown area of the wing into consideration. The average value for this second phenotype is 184.4 with a standard deviation of 42.4 compared to the average value of 0.71 with a standard deviation of 0.18 for the single feather analysis used for the eQTL analysis (see below).

**Figure 3:**
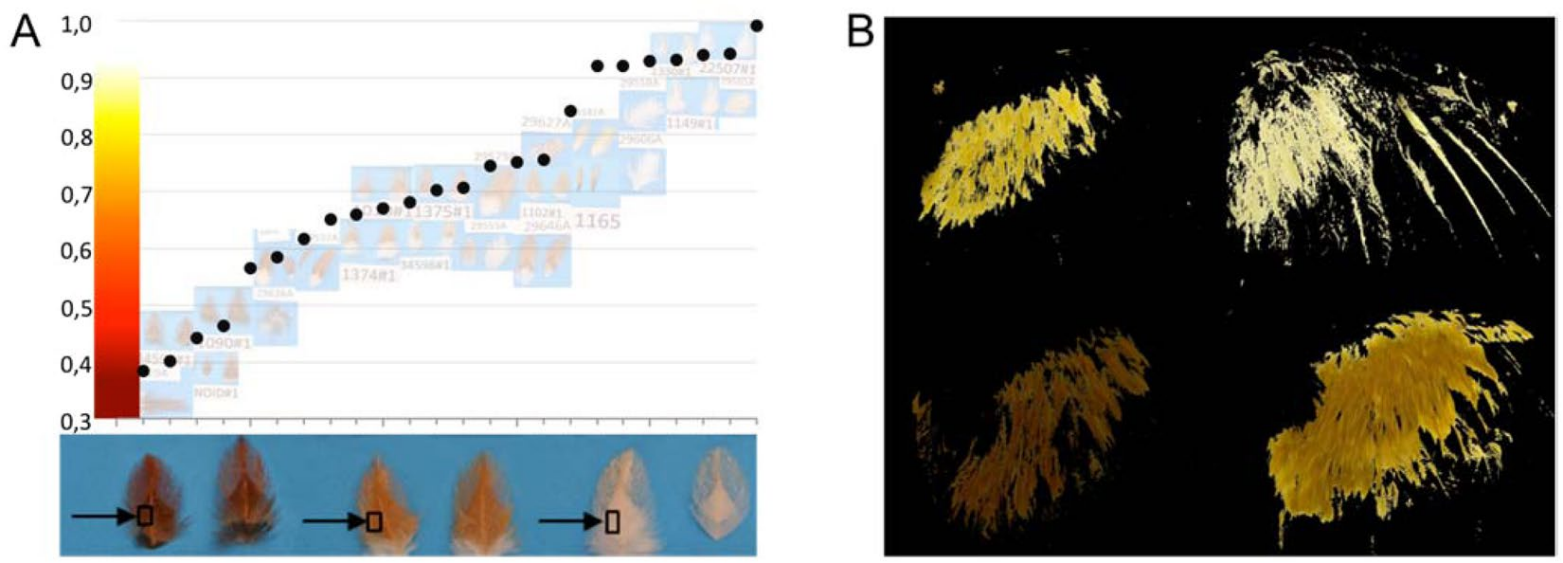
Quantitative colour phenotypes used for QTL/eQTL mapping. (**A**) Peak red intensity. The feathers that we have used in the gene expression measurements are placed on a blue background and their level of red intensity is measured relative to a True red colour chart. The darkest red feathers score between ~0.4–0.5 and the almost pure white feathers score between ~0.9–0.99. (**B**) Depicts four representative measurements of Average red colour score measured across the right wing.

A separate eQTL fine mapping population was used for expression analysis of budding feather tissue for candidate gene assessment in the previously detected QTL regions identified in the F_8_ generation. These birds were individuals from both the F_10_ and F_12_ generations from the advanced intercross (n = 12 F_10_ and 12 F_12_ birds). Multiple generations were required to obtain a broad spectrum of red colour phenotypes, see Fig. 3, with colours ranging from dark red to light orange. Only birds with some degree of red-brown colouration were used in this study, with all these birds (n = 24) genotyped and phenotyped as below. For these birds, growing feather buds were selected from adult wings for use in both phenotyping and gene expression assays. These feather buds were selected from cover feathers in the central areas of each wing. The feathers from each growing bud were removed and used in the phenotyping assay, to ensure that the gene expression and colour phenotype would match as closely as possible. These feathers were removed from the bud and stacked in 3 layers to mimic the wing (where feathers are overlapping). Red colour intensity was then measured using the same technique as for the F_8_ birds, with only the peak intensity measured. The developing basal root of the feather follicle (calamus) was then used for RNA extraction and microarray analysis for eventual eQTL analysis.

### RNA extraction

5–6 growing feathers of 2–3 cm length were collected from each individual. The calamus was immediately cut from each feather and snap frozen in liquid nitrogen for RNA extraction, the rest of the feather was kept at −30 and used for colour measurements. RNA was extracted from a pooled sample of three to four calami per individual which had been homogenized in Lysing matrix D tubes(MP Biomedicals) in a FastPrep(MP Biomedicals) using TRIzol (Sigma Aldrich) reagent and following the manufacturers instructions. After quality checking the RNA with RNA Nano Chips(Agilent Technologies) in an Agilent 2100 Bioanalyzer to ensure that RNA was not degraded, the RNA was treated with DNAse I (Thermo scientific) followed by cDNA synthesis using a RevertAID KIT from Life technologies, and following the instructions provided with the kit. The cDNA was again quality checked using a Bioanalyzer 2100 and RNA Nano chips (Agilent Technologies) and subsequently labeled with a NimbleGen One colour labeling kit(Roche) according to the manufacturer’s instructions. Finally, the cDNA was hybridized to NimbleGen 12 × 135 k custom gene expression microarrays (Roche) and scanned using a NimbleGen MS200 Microarray scanner (Roche). The custom Microarrays have been used in previous publications by the group and contain all known Ensembl and RefSeq genes as well as EST probes (for microarray details see^62^). Microarrays were first preprocessed using DEVA software to normalize the data and all arrays were processed together.

### Genotyping, QTL mapping

DNA preparation was performed by Agowa GmbH (Berlin, Germany), using standard salt extraction. A total of 652 SNP markers were genotyped using an Illumina GoldenGate system, and gave a map of length ~92675 cM, with an average marker spacing of ~16 cM. Of these markers, 551 were fully informative (differentially fixed between the parental populations), with markers selected to be evenly spread throughout the genome (as reflected in the average marker spacing) and based around the markers generated for the F_2_ mapping of this intercross^63,64^. The QTL analysis was performed in R^65^ using the R/Qtl software package^66^, with standard interval mapping and epistatic analyzes performed. Interval mapping was performed using additive and additive + dominance models. In the colour phenotyping QTL analysis batch and sex were always included as fixed effects. To control for potential family substructure, a Principal Component Analysis (PCA) of the genotype data was performed, and the first ten PCs fitted as covariates in the QTL model, with all significant PCs retained in the final model (see^62^ for further details). A sex interaction was also added, where significant. Digenic epistatic analysis was performed as per the guidelines provided in^67^. Initially a global model was used that incorporated standard main effects and sex interactions. Epistasis was then built on this model, starting with the most significant loci and working down for each trait. Significance thresholds were calculated by permutation^68,69^, with thresholds of 20% and 5% genome wide significance being the cut-offs for suggestive and significant loci respectively. A suggestive threshold was used as per^70^, though we used a 20% genome wide threshold, which is slightly more stringent than 1 false positive per scan. These thresholds corresponded to approximately LOD cut offs of 3.6 and 4.4. Confidence intervals were calculated using a 1.8 LOD drop method^71^. Epistasis thresholds were calculated in a similar manner, with 20% and 5% genome-wide thresholds used. If two loci were significant by main effects alone then an epistatic effect for this locus was included if the interaction passed the nominal 5% threshold in the same manner as a sex interaction. Note that the sample sizes required to detect epistatic interactions can be much larger than a standard QTL analysis (relying on different allelic combinations that may have relatively low proportions in the intercross), so certain interactions will most likely be missed, though ones that are detected will still be valid.

### Functional validation Via targeted eQTL mapping

The individuals in the F_10_ and F_12_ fine mapping eQTL population were genotyped for sixteen SNP markers which had been identified in the F_8_ mapping population as being flanking markers for the QTL with the two colour phenotypes (see Supplementary Table 1 for locations and primers). In this manner, a targeted scan aimed specifically at the colour QTL could be performed. Due to the targeted nature (i.e. the fact only a subset of genes were analysed), the significance threshold was far lower than for a full genome scan. Five were located on chromosome 2, three on chromosome 10 and chromosome 14, and two markers on chromosome 15 and chromosome 11 and finally one marker on chromosome 26, for a total of 16 markers. These SNP markers were all genotyped by pyrosequencing on a QIAGEN pyromark q24 using QIAGEN GOLD reagents. DNA was extracted from blood using a standard salt extraction protocol, except for a small number of individuals were blood samples were missing and DNA was instead extracted from feathers using a modified protocol for the Qiagen DNEasy kit with dithiothreitol added to the lysis step.

Analysis was performed using a three-step procedure. Initially, all genes that were present in the colour QTL regions detected in the F_8_ analysis were selected as candidates (n = 875, Reference genome version GalGal4). These probes were then correlated with the specific red colour phenotype obtained for each feather sample (see above), using a linear model to fit gene expression levels and colour score using the lmFit function in the limma R package^72,73^. All probes that had a significant association between gene expression and colour score at a FDR significance level of 0.05 were retained (n = 76, see Supplementary Table 2). The last step was to perform eQTL mapping in R/qtl, using standard interval mapping, with sex and batch (F_10_ or F_12_) included as fixed factors. All the genes from second step were tested against the regions genotyped using the 16 SNP markers mentioned above (on chromosomes 2, 10, 11, 14, 15 and 26). Permutation testing was used to set an experiment-wide threshold for each gene (1000 permutations per gene), with a 5% significance and a 20% suggestive threshold obtained (LOD values of ~3.0 and ~2.2 respectively). To control for the total number of genes (n = 76), a principal component analysis was first performed on these 76 genes. 7 significant eigenvalues accounted for 97% of the total variation in the data. Thus, as it is only required to control for the number of independent tests, a multiple testing correction of 7 was added to each LOD threshold. Using a log_10_ transformation to convert this to a LOD score, this gives an increase of 0.85 LOD to each threshold, meaning an eQTL was suggestive with a LOD score of ~3.05, and significant with a LOD score of ~3.85.

## Supplementary information

Supplementary Information.

## Data Availability

Genotype data is available at the following link on figshare with the following doi: 10.6084/m9.figshare.125060. Microarray data for the feathers is available on ArrayExpress with the following accesion number: E-MTAB-8689.
