# Supplementary material for: CREBBP and WDR 24 Identified as Candidate Genes for Quantitative Variation in Red-Brown Plumage Colouration in the Chicken
Source: Sci Rep. 2020 Jan 24;10:1161. doi: 10.1038/s41598-020-57710-7 (PMC6981141; doi:10.1038/s41598-020-57710-7)
Supplement: Supplementary file 1 — Supplementary Information. [file 41598_2020_57710_MOESM1_ESM.pdf]

CREBBP and *WDR 24* Identified as Candidate Genes for Quantitative Variation in Red-Brown Plumage Colouration in the Chicken

Fogelholm, J.[1], Henriksen. R.[1], Höglund, A.[1], Huq, N.[1], Johnsson, M.[3,4], Lenz, R.[2], Jensen, P.[1], Wright, D.[1] *

SUPPLEMENTARY INFORMATION

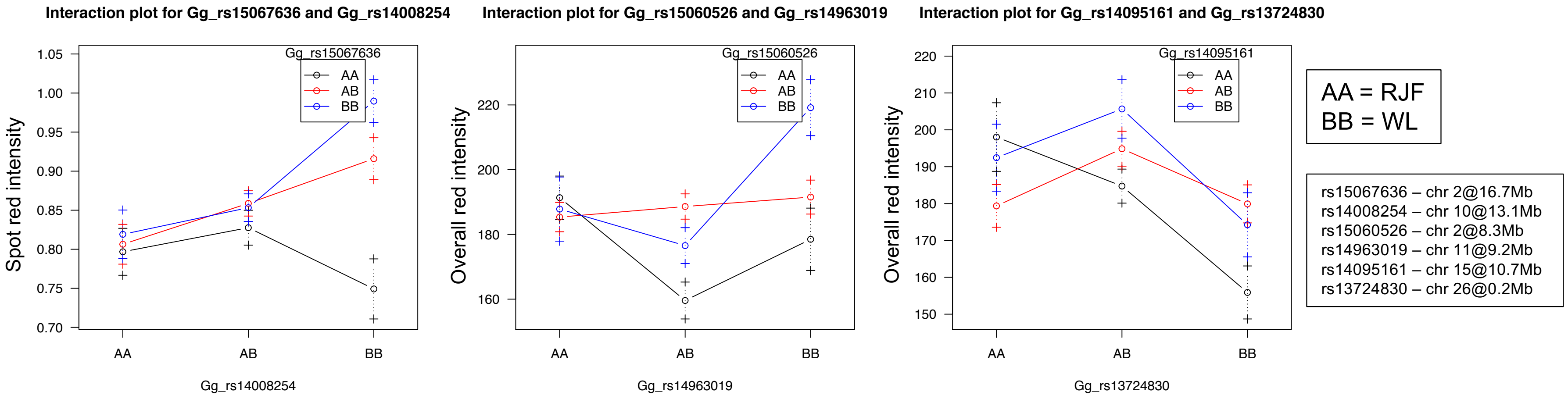

Supplementary Figure 1. Epistatic interactions present in colour QTL (spot and overall red intensity traits).

trait	epistatic combination	estimate	SE	t
overall_red_intensity	2@81.0add:11@73.0add	13.1	4.6	2.8
overall_red_intensity	2@81.0dom:11@73.0add	-4.74	6.56	-0.72
overall_red_intensity	2@81.0add:11@73.0dom	-7.38	6.54	-1.13
overall_red_intensity	2@81.0dom:11@73.0dom	42.59	9.50	4.48
overall_red_intensity	15@148.0add:26@0.0add	16.84	5.35	3.15
overall_red_intensity	15@148.0dom:26@0.0add	34.60	10.06	3.44
overall_red_intensity	15@148.0add:26@0.0dom	5.42	7.70	0.70
overall_red_intensity	15@148.0dom:26@0.0dom	-20.45	14.81	-1.38
spot_red_intensity	2@149.0add:10@177.0add	0.06	0.02	3.18
spot_red_intensity	2@149.0dom:10@177.0add	0.08	0.03	2.21
spot_red_intensity	2@149.0add:10@177.0dom	-0.07	0.03	-2.39
spot_red_intensity	2@149.0dom:10@177.0dom	-0.02	0.05	-0.51
spot_red_intensity	10@177.0add:sex	-0.08	0.02	-3.28
spot_red_intensity	10@177.0dom:sex	-0.02	0.04	-0.59

Supplementary Table 1. Expansion of epistatic interactions observed in the colour QTL. ‘Add’ and ‘Dom’ refers to whether the effect was additive or dominant in each specific pair, with additive x additive, additive x dominance, dominance x additive, and dominance x dominance interactions shown, with the effect estimate, standard error and t-value given.

trait	chr	position	LOD	R2	add+/-s.e.	dom+/-se	lower CI	upper CI	lower_marker	upper_marker	covariates	interaction
spot_red_intensity	2	180	4.2	3.9	0.06+/-0.01	0.06+/-0.02	95	199	rs15060526	2_23979784	sex, batch, PC1, PC3	
spot_red_intensity	10	205	4.8	4.6	0.20+/-0.04	0.02+/-0.07	188	233	rs14949856	rs14952325	sex, batch, PC1, PC3	10@176.0:sex
spot_red_intensity	14	206	3.9	3.6	-0.07+/-0.02	-0.05+/-0.03	163	228	rs14076550	rs15002638	sex, batch, PC1, PC3	

Supplementary Table 2. QTL effects without including any epistatic interactions for the peak colour QTL loci.
